# Roux-en-Y gastric bypass in a transgender patient: a case report

**DOI:** 10.1093/jscr/rjae168

**Published:** 2024-03-17

**Authors:** Rahul Menon, Phil Lockie

**Affiliations:** General Surgery Department, Ipswich Hospital, Ipswich QLD 4305, Australia; General Surgery Department, Ipswich Hospital, Ipswich QLD 4305, Australia

**Keywords:** bariatric surgery, transgender, gender reassignment surgery, multidisciplinary care

## Abstract

The frequency of transgender individuals seeking gender affirming care is increasing over the last decade. Transgender patients suffer from obesity and psychiatric illness at elevated levels compared with the general population. A 54-year-old male-to-female transition patient presented with morbid obesity, hyperlipidaemia, and weight gain 2 years after their gender-transition and hormonal therapy. She received a Roux-en-Y gastric bypass (RYGB). At 7 months postoperatively, the patient has experienced 49% excess body weight loss, her body dissatisfaction had resolved and has completed further plastic surgery. The RYGB is an effective method for weight loss as well as benefits from cardiovascular disease, cancer, metabolic related conditions, and psychosocial wellbeing. Holistic treatment in conjunction with dietetic and psychology services can help maintain long-term weight management. Bariatric surgery combined with a multidisciplinary care team addressing medical and psychiatric concerns is integral to achieving and maintaining weight loss and gender identity.

## Introduction

Previously, medical procedures were the only treatment for transgender individuals seeking gender-affirming care; however, over the past 10 years, both medical and surgical care options have been increasing [1–3]. Gender-affirming surgery is being performed at greater rates worldwide comprising in a number of treatments to cope with psychological, emotional, and physical stresses with gender affirmation [4].

Obesity is far more prevalent in the transgender population [3] with over 25% of transgender individuals experiencing obesity with a further proportion seeing rises in BMI after initiation of hormone therapy [5]. In addition to this, higher BMI prevents many transgender individuals engaging with gender-affirming surgical procedures [6]. Furthermore, the prevalence of eating disorders and psychosocial problems linked to body dysmorphia and dissatisfaction co-exist at higher levels in the transgender population [7, 8, 3]. This may co-exist with unwanted features of their natal gender and make weight loss and management especially difficult [3].

For the morbidly obese, bariatric surgery remains the only long-lasting and effective weight-loss option. This case highlights the use of a Roux-en-Y gastric bypass (RYGB) to assist a male-to-female (MTF) transgender patient to express their gender identity and combat the weight gain and metabolic side effects from hormonal therapy as well as important considerations in pre- and post-operative care pertaining to bariatric surgery and transgender individuals.

## Case report

This case features a 54-year-old Caucasian MTF transgender patient who had undergone gender transition surgery with a robotic assisted combined peritoneal pull through and penile inversion surgery in 2021 and has been enrolled in hormonal therapy since. She had been struggling with her weight since surgery and hormonal therapy and had gained 35 kg in weight over the last 18 months. Due to her weight gain she was struggling to complete her gender transition surgery and was suffering from hyperlipidaemia as well as body image dissatisfaction from weight gain. She obtained a referral to a specialist bariatric clinic seeking further surgical options. She did not have any other medical conditions. Her pre-operative weight was 135 kg and had a BMI of 40.3 kg/m^2^, her total cholesterol was elevated at 6.2 mmol/L (with elevated LDL at 4.21 mmol/L), all her other blood tests and parameters were normal.

Following routine multidisciplinary (surgeon, dietician, clinical psychologist) assessments, bariatric surgery was recommended due to ongoing obesity and evidence of metabolic disease. She underwent a screening gastroscopy, which was normal and following this a RYGB. During surgery, a vertical gastric pouch was created with a powered laparoscopic stapler and a 150 cm biliopancreatic limb was fashioned after this, a gastrojejunostomy was created with a 75 cm Roux limb. There were no postoperative medical complications and she attended dietetic and psychology follow-up along with standard surgical follow-up. She experienced steady weight loss and at 7-months follow-up had 49% excess body weight loss (%EWL) giving her a BMI of 34 kg/m^2^. She also had improvement in her cholesterol with a total cholesterol of 5.2 mmol/L (and LDL of 3.72 mmol/L).

She reported functional improvements in her physical mobility and was engaging in exercise and was also recently able to complete her plastic surgery. She reported improved confidence, body image satisfaction and was engaging regularly with psychology services and group therapy.

## Discussion

The prevalence of obesity continues to grow in Australia, as does morbid obesity [9]. Obesity affects both sexes, however, there is greater prevalence in transgender populations [3] with a higher risk of poor physical and mental health and disability compared with non-transgender population [7, 8]. This highlights the importance of psychosocial support in the management of those with gender dysmorphia and champions a multidisciplinary approach when managing body weight [3, 10]. Counselling should be considered prior to the initiation of hormonal therapy and in the case of the morbidly obese, bariatric surgery is beneficial [3].

RYGB is a restrictive-malabsorptive operation performed for weight loss in patients with morbid obesity (BMI > 40 kg/m^2^) or those who have BMI >35 kg/m^2^ with obesity related or metabolic comorbidities [11]. It is one of the most popular options offered for bariatric surgery in Australia, and long considered the ‘gold standard’ in bariatric surgery [12]. Large prospective studies on bariatric surgery have indicated that maximal weight loss is achieved between 12 and 24 months postoperatively, a fifth of these patients maintain weight loss at 20 years [13]. In general, RYGB offers the largest mean percentage weight loss (25–35%), followed by sleeve gastrectomy (20–30%) and adjustable gastric band (17–20%) [12]. Bariatric surgery also improves mortality from cardiovascular disease, cancer and reduces metabolic related complications [14]. Marked weight loss from bariatric surgery also vastly improves glycaemic control and improves other modifiable cardiovascular risk factors [15]. In our case, we observe that the patient experienced 49% EWL with ongoing weight loss and improvement in metabolic parameters.

Those who wish to undergo bariatric surgery often suffer from a lower quality of life as well as other psychosocial conditions such as depression anxiety and eating disorders [16]. Similarities in the lifetime rates of mood disorders and self-harm rates (5 vs. 36%) between bariatric surgery candidates and transgender individuals [17, 18] necessitate thorough psychological evaluation and suicide risk assessment [19]. The use of hormonal therapy has undesirable medical side effects but notably risks weight gain and secondary sexual characteristics [16]. Therefore, a holistic approach comprising psychologists, dietitians, and doctors helps patients with the physical and psychological stresses of surgery and weight loss and ensures that patients continue to maintain positive, healthy habits. Counselling on hormonal side effects and endocrinology input is also beneficial.

## Conclusion

Both bariatric surgery candidates and transgender people exhibit higher rates of psychiatric illness and suicidality and would benefit from a collaborative, multidisciplinary team approach pre- and post-operatively. The use of hormone therapy can indirectly and directly affect post-surgical weight loss and associated metabolic conditions. No studies to date have examined weight loss patterns amongst transgender individuals who are undergoing bariatric surgery or the concomitant effect of hormone therapies highlighting areas of further study.
